# The microRNA-381(miR-381)/Spindlin1(SPIN1) axis contributes to cell proliferation and invasion of colorectal cancer cells by regulating the Wnt/β-catenin pathway

**DOI:** 10.1080/21655979.2021.2003663

**Published:** 2021-12-27

**Authors:** Ling Zhou, Heng Wang, Zhi Fang, Min Zhong, Yan He, Jianping Zou, Shanshan Huang, Junhe Li, Xiaojun Xiang, Ziling Fang

**Affiliations:** a Department of Oncology, The First Affiliated Hospital of Nanchang University, Nanchang, Jiangxi Province, P.R. China; b Department of Orthopedics, The First Affiliated Hospital of Nanchang University, Nanchang, Jiangxi Province, P.R. China

**Keywords:** SPIN1(Spindlin1), miR-381(microRNA-381), colorectal cancer, proliferation, invasion

## Abstract

Our study aimed to investigate the clinical significance and biological functions of Spindlin1 (SPIN1) in colorectal cancer (CRC) tumorigenesis and progression, as well as the mechanism underlying its upregulation. The expression of SPIN1 was detected by immunohistochemistry and western blotting assays. Bioinformatics prediction and dual-luciferase reporter assays were used to determine whether microRNA-381 (miR-381) could target SPIN1. A series of cell functional experiments were performed to investigate whether the miR-381-mediated regulation of SPIN1 is involved in the progression and aggressiveness of CRC cells via the Wnt/β-catenin pathway. Our results showed that SPIN1 is frequently overexpressed in CRC tissues and cell lines, and its upregulation is positively correlated with disease progression and lymph node metastasis. Moreover, SPIN1 depletion suppresses cell growth, migration, and invasion through inactivation of the Wnt/β-catenin signaling pathway, which recapitulates the effects of miR-381 upregulation. Moreover, SPIN1 is a target gene of miR-381, and miR-381 is downregulated in CRC. Furthermore, the reintroduction of SPIN1 partially abolished the miR-381-mediated inhibitory effects in CRC cells. In summary, our data revealed that the miR-381/SPIN1 axis greatly contributes to CRC tumorigenesis by orchestrating the Wnt/β-catenin pathway, thereby representing actionable therapeutic targets for colorectal cancer patients.

## Introduction

Colorectal cancer (CRC) is one of the leading causes of cancer mortality worldwide, with a high incidence, rapid progression, and great metastatic potential [1]. Despite the numerous improvements in surgery, chemotherapy, radiotherapy, and immunotherapy, the overall survival of patients with CRC remains dismal [2]. At present, it is of great importance to uncover the complex underlying mechanisms responsible for CRC carcinogenesis and progression.

Spindlin1 (SPIN1), a member of the SPIN/SSTY family, was first identified as an overexpressed protein in ovarian cancer [3]. The Chromatin reader SPIN1 recognizes trimethylated histone H3 lysine 4 to facilitate rRNA expression via protein-protein interaction [4]. Recently, several studies have defined the oncogenic role of SPIN1 in tumorigenesis and progression in several cancers, such as non-small cell lung cancer (NSCLC), liver cancer, gastric cancer, and breast cancer [5–8]. Our previous study identified SPIN1 as a novel and negative regulator of the uL18-MDM2-p53 pathway to control cancer cell proliferation and apoptosis [9]. Additionally, the Wnt, PI3K/AKT, and RET pathways regulated by SPIN1 during tumorigenesis have been reported [10]. Nevertheless, the cellular functions and clinical significance of SPIN1, along with the mechanism underlying its dysregulation in colorectal cancer, remain poorly characterized.

Emerging evidence has shown that miRNAs, a group of non-coding RNA molecules containing 20 to 22 nucleotides, were found to be involved in multiple physiological and pathologic processes [11,12], such as cell differentiation, invasion, and tumorigenesis [13–15]. Specifically, the interaction between miRNAs and oxidative stress signals is involved in gastrointestinal carcinogenesis [16]. Past studies have demonstrated that several miRNAs have been shown to contribute greatly to the development and progression of colorectal cancer [17–20], among which miR-381 was discovered to exert tumor-suppressive functions. In addition, our preliminary bioinformatic predictions indicated that miR-381 could bind to the 3ʹ-UTR of SPIN1 mRNA. However, the exact correlation between miR-381 and SPIN1 in CRC has not been specifically reported.

Therefore, we hypothesized that the miR-381-mediated regulation of SPIN1 plays a significant role in the tumorigenesis and progression of CRC. In this study, our data showed that miR-381 inactivates the Wnt/β-catenin signaling pathway by targeting SPIN1, and therefore represents an actionable therapeutic target for colorectal cancer patients.

## Materials and methods

### Ethics statement and clinical tissues

Primary colorectal cancer tissues (from 2019 to 2020) and paraffin-embedded colorectal cancer samples (from 2016 to 2018) were obtained from the First Affiliated Hospital of Nanchang University. After resection, the fresh specimens were frozen and immediately stored in liquid nitrogen. Detailed clinicopathological features of the patients were collected and listed in Supplementary Table S1 and Table 1. None of the patients with colorectal cancer received radiotherapy, chemotherapy or immunotherapy before surgery. Our study was approved by the First Affiliated Hospital of the Nanchang University Ethics Review Board and complied with the Declaration of Helsinki. All CRC patients who participated in this study signed an informed consent document.

**Table 1.** The relationship between SPIN1 expression and clinical features of colorectal cancer patients

		SPIN1 status (%)		P value
Clinicopathological factors	N	Negative	Positive	
	90	37 (41.1)	53 (58.9)	
Age (years)				
<55	48	20 (41.7)	28 (58.3)	0.909
≥55	42	17 (40.5)	25 (59.5)	
Sex				
Male	44	20 (45.5)	24 (54.5)	0.413
Female	46	17 (37.0)	29 (63.0)	
Tumor Size (cm)				
<5	42	16 (38.0)	26 (62.0)	0.586
≥5	48	21 (43.8)	27 (56.2)	
Depth of invasion				
T_1_-T_2_	30	18 (60.0)	12 (40.0)	0.01
T_3_-T_4_	60	19 (31.7)	41 (68.3)	
Lymph node metastasis				
With	38	9 (23.7)	29 (76.3)	0.004
Without	52	28 (53.8)	24 (46.2)	
TNM stage				
I–II	46	26 (56.5)	20 (43.5)	0.002
III–IV	44	11 (25.0)	33 (75.0)	

All the data were analyzed by the chi-square test.

### Cell culture

The human colon epithelial cell line, NCM460 (Cat. No. CRL-1831), CRC cell line DLD1(Cat. No. CCL-221), SW48 cells (Cat. No. CCL-231), SW480 (Cat. No. CCL-228), LOVO (Cat. No. CCL-229), HT-29 and HCT116 were obtained from the American Type Culture Collection (ATCC) or donated by Sun Yat-sen University. All cells were cultured in Dulbecco’s modified Eagle’s medium (DMEM, HyClone, Logan, UT, USA) supplemented with 12% fetal bovine serum (FBS; HyClone, USA) and penicillin-streptomycin antibiotics at 37°C and 5% CO_2_.

### Cell transfection

HCT116, LOVO and DLD1 were selected for our further analysis due to the different expression levels of SPIN1. The negative control siRNA, SPIN1 siRNAs, NC mimics, miR-381 mimics, NC inhibitors, miR-381 inhibitors and SPIN1 overexpression plasmids were designed and synthesized by GenePharma (Suzhou, China). SPIN1 expression vector without 3′-UTR (SPIN1-no UTR) was constructed by inserting its CDS sequence into the vector (Promega, USA). All the transfection experiments were performed using Turbofect Reagent (Thermo Fisher, St Louis, MO, USA) according to the manufacturer’s instructions. The indicated cells were harvested 48 h upon transfection. Transfection efficiency was confirmed by qRT-PCR (quantitative real-time PCR) and western blotting.

### Cell counting kit-8 (CCK-8) assay

Indicated cells were inoculated at 1 × 10^3^ cells per well in 96-well plates for 1, 2, 3, 4, and 5 days. After adding 10 μL of the CCK-8 reagent post-transfection at the same time every day, the absorbance values of treated cells were detected daily at 450 nm using a Microplate Reader (Molecular Device, SpecrtraMax M5e, Sunnyvale, CA, USA) for five consecutive days.

### Colony formation assays

In this study, 1 × 10^3^ transfected CRC cells (LOVO, DLD1, and HCT116) were seeded into 6-well plates. After 10–15 days of incubation, the colonies were processed with paraformaldehyde and crystal violet for 60 min. ImageJ software was used to count the visible colonies. All experiments were performed at least twice.

### Scratch wound and transwell invasion assays

Scratch wound and transwell invasion assays were performed to evaluate cell migration and invasion. The same number of indicated cells were seeded on a 6-well plate, and the cells were allowed to adhere to the plate and reach full confluency. A sterile 10 μL pipette tip was used to draw straight lines to make a wound. Microscopes were utilized to take images at the indicated times for further analysis.

Indicated cells were resuspended and plated into the upper chamber (8.0 μm pore size; Corning, USA). Upon incubation, the number of cells that invaded the bottom was estimated using the average number of cells over three microscopic fields. Biological experiments were performed more than twice.

### Dual-luciferase reporter assay

Dual-luciferase reporter assays were carried out as reported previously [21]. The binding sites between miR-381 and SPIN1 were mutated, and the SPIN1 mutant plasmid was constructed for the dual luciferase reporter assay. Briefly, the vector containing wild-type (WT) or mutated (MUT) 3ʹUTR of SPIN1 and miR-381 mimics/miR-381 inhibitor or negative control were transfected into CRC cells using TurboFect Reagent (Thermo Fisher, St Louis, MO, USA). Thirty-six hours post-transfection, the indicated cells were harvested and analyzed using a propriate luciferase detection kit (E1910, Promega, Madison, WI, USA). Luciferase activity was normalized to Renilla luciferase activity.

### Western blotting analysis

Western blotting analysis was performed as previously described [21,22]. The antibodies and concentrations utilized in our study were shown as follows: SPIN1 (1:1500; #12,105-1-AP, Proteintech, Wuhan, China), β-catenin (1:1000; #8480, Cell Signaling Technology, MA, USA), cyclinD1 (1:1000; #55,506, Cell Signaling Technology, MA, USA), c-Myc (1:1000; #18,583, Cell Signaling Technology, MA, USA), β-actin (1:2000; #AF7018; Affinity, Jiangsu, China), and GAPDH (1:4000; #A2220; Affinity, Jiangsu, China). β-actin and GAPDH were used as internal controls, and the immunoblotting bands were visualized using ImageJ software. More than two independent experiments were performed.

### Immunohistochemistry (IHC) assays

IHC was performed on 90 clinical CRC specimens. Briefly, the paraffin-embedded tissue was cut into 5 µm-thick tissue sections, which were then baked and fixed, deparaffinized with xylene, hydrated with ethanol, and heated in an antigen retrieval solution (EDTA, pH 9.0), after which endogenous peroxidase was quenched with 3% H_2_O_2_. After blocking with goat serum, the samples were incubated with anti-SPIN1 antibody (1:50, #19,531-1-AP, Proteintech, Wuhan, China) at 4°C overnight. On the second day, the tissue was covered with HRP-conjugated secondary antibody for 30 min and stained with 3,3ʹ-diaminobenzidine until brown particles appeared in the membrane, cytoplasm, or nucleus. Finally, the sections were counterstained with hematoxylin at room temperature. SPIN1 protein expression in CRC samples was determined using the staining index (SI). Samples with SI ≥ 6 were determined to have high SPIN1 expression, whereas those with SI < 6 were shown as low SPIN1 expression. SPIN1 immunostaining scores were evaluated by two experienced pathologists.

### Quantitative real-time PCR (qRT-PCR) assays

Total RNA from transfected cells was collected using TRIzol (Invitrogen, Carlsbad, CA, USA), and qRT-PCR assays were performed using the SYBR Green PCR kit (BioRad, Hercules, USA) according to the manufacturer’s instructions. The fold changes in mRNA expression among the groups were assessed using the 2^–∆∆Ct^ method. The sequences of the primers used are listed in Table 2. U6 and GAPDH were used to normalize the variation. All experiments were performed three times.

**Table 2.** The sequences of primers for qRT-PCR used in this study

Gene	Primers
SPIN1	forward primers: GGTATCCAGGTGCCTTATG reverse primers: GGACGATCTGCGACTATTG
miR-381	forward primers: TGGTACTTAAAGCGAGGTTGC reverse primers: GGTCATGCACACACATACCAC
U6	forward primers: CTCGCTTCGGCAGCACA reverse primers: AACGCTTCACGAATTTGCGT
GAPDH	forward primers: GATTCCACCCATGGCAAATTC reverse primers: AGCATCGCCCCACTTGATT

### Statistical analysis

All statistical analysis was performed using SPSS 20.0 software. These biofunctional experiments were repeated more than 2 times. Analysis of the correlations between SPIN1 or miR-381 and clinical features in CRC patients was performed using Chi-square tests. Statistical significance among groups was analyzed by one-way analysis of variance (ANOVA) or by the Student’s two-tailed t-test. Differences were considered statistically significant at P values lower than 0.05.

## Results

### SPIN1 is upregulated in CRC tissues and cell lines

**Figure 1.**
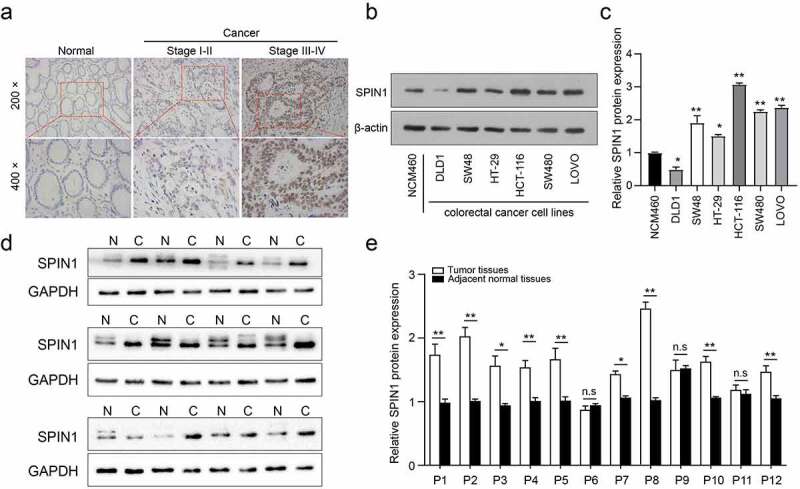
SPIN1 is upregulated in CRC tissues and cell lines. (a) Immunohistochemical staining images of SPIN1 in 90 pairs of paraffin-embedded colorectal cancer (CRC) tissues and adjacent non-cancerous tissues (magnification, 200× and 400×). (b, c) Representative western blotting images and quantitation of SPIN1 protein expression in six CRC cell lines and normal colon cells. (d) The SPIN1 protein expression in 12 pairs of CRC tissues and adjacent normal tissues were assessed by western blotting analysis. (e) The quantification analysis of the blots was analyzed by image J software. P, patient; Student’s t test, *p < 0.05; **p < 0.01.

Our previous data have demonstrated that SPIN1 functions as a negative modulator of the uL18-MDM2-p53 signaling pathway, thus promoting tumorigenesis in human cancer [9], but its role in CRC remains largely unknown. In this study, our immunohistochemical staining of 90 patients showed that SPIN1 was mainly localized in the nucleus of CRC cells, with higher SPIN1 expression in CRC specimens when compared to paired non-cancerous tissues (53/90, 58.9%, illustrated in Figure 1(a)). Moreover, the protein expression of SPIN1 was higher in most CRC cell lines than in the normal colon epithelial cell line NCM460 (Figure 1(b,c)). Furthermore, we analyzed the correlation between SPIN1 expression and clinicopathological factors in the CRC cohort (n = 90). As shown in Table 1, SPIN1 expression was strongly correlated with depth of invasion (p = 0.01), clinical TNM stage (p = 0.002), and lymph node metastasis (p = 0.004). Nevertheless, no statistically significant associations were found between SPIN1 expression and age, sex, or tumor size. Consistently, the protein expression of SPIN1 in fresh CRC tissues was also upregulated (9/12, 75%, Figure 1(d,e)). Our data demonstrated that SPIN1 was markedly elevated and positively correlated with disease progression in CRC.

### SPIN1 depletion inhibits cell proliferation, migration and invasion

**Figure 2.**
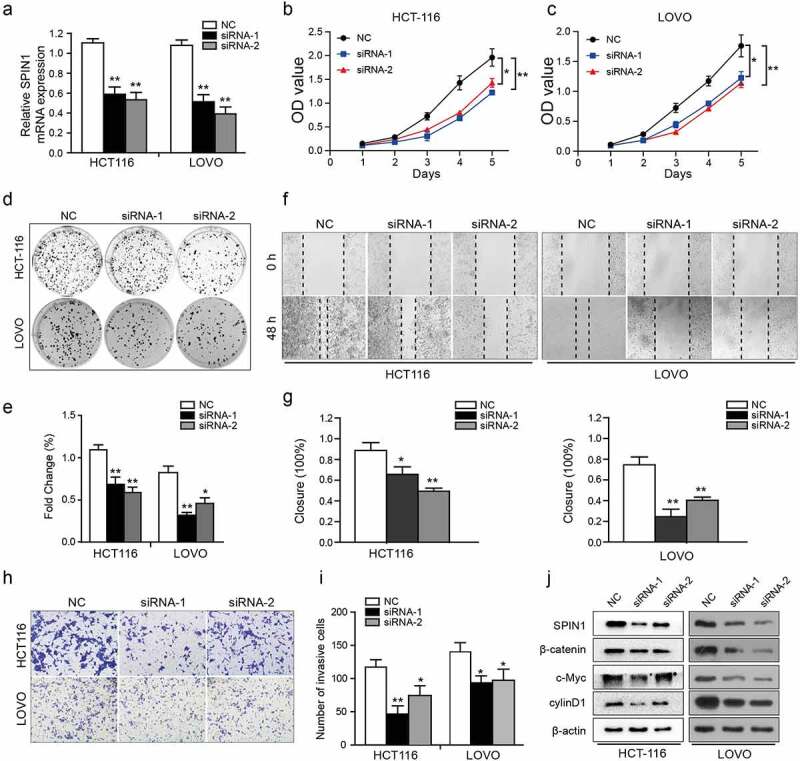
SPIN1 depletion inhibits cell proliferation, migration and invasion. (a) qRT-PCR assays were utilized to confirm the transfection efficiency of siRNAs in HCT-116 and LOVO cells. (b, c) Cell proliferation ability was tested by CCK-8 assays. (d, e) The colony forming assays were used to evaluate the colony formation ability. (f- i) The migration and invasion abilities were evaluated by scratch wound assays (f, g) and transwell invasion assays (h, i) upon transfection of negative control siRNAs and SPIN1 siRNAs, respectively. (j) Representative western blotting analysis of SPIN1 and Wnt/β-catenin pathway components in HCT-116 and LOVO cells upon indicated transfection. Student’s t test, *P < 0.05 and **P < 0.01.

As SPIN1 overexpression was markedly correlated with aggressive clinical features of CRC patients, therefore, we further utilized a number of cellular functional experiments to investigate the biological role of SPIN1 in CRC cells. After transfection, the efficiency of siRNA transfection was assessed by qRT-PCR and western blotting simultaneously (Figure 2(a,j)). Subsequently, CCK-8 and colony forming assays showed that SPIN1 depletion significantly suppressed the cell growth of CRC cells (Figure 2(b-e)). Moreover, SPIN1 downregulation resulted in decreased cellular migration and invasion abilities (Figure 2(f-i)). Mechanistically, SPIN1 knockdown reduced the protein expression of β-catenin, c-Myc and cyclinD1, which are the core components of the Wnt pathway (Figure 2(j)). Collectively, our results indicate that SPIN1 plays a critical role in accelerating cell proliferation and invasion by positively regulating the Wnt/β-catenin pathway.

### SPIN1 is a target of miR-381 and miR-381 is downregulated in CRC

**Figure 3.**
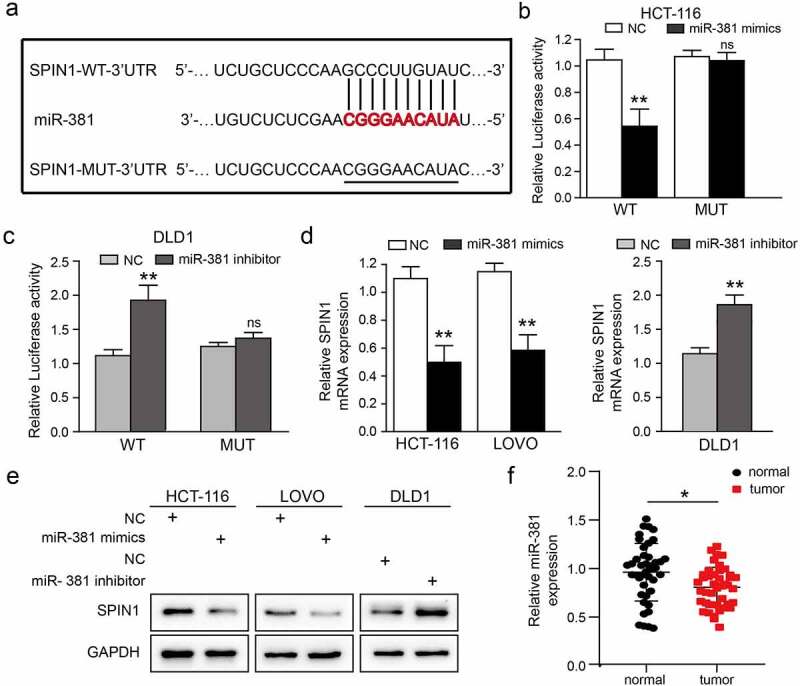
SPIN1 is a target of miR-381 and miR-381 is downregulated in CRC. (a) Construction of the Wild-type (WT) and mutated (MUT) SPIN1 3ʹUTR luciferase reporter vectors. (b, c) The dual-luciferase reporter kit was used to detect the luciferase activity of WT and MUT SPIN1 3ʹUTR plasmids in HCT116 cells and DLD1 cells upon indicated transfection, respectively. (d) The expression of SPIN1 mRNA was assessed by qRT-PCR assays after indicated transfection in CRC cells. (e) Western blotting analysis of SPIN1 protein in CRC cells upon transfection by miR-381 mimics, miR-381inhibitor or negative controls. (f) Relative expression of miR-381 in colorectal cancer (CRC) tissues and paired adjacent tissues (n = 42). Student’s t test, *p < 0.05, **p < 0.01.

Bioinformatic tools (TargetScan and miRanda) were used to screen and identify potential miRNAs that could target SPIN1 in our study. We finally selected miR-381 for further investigation, as it was projected by these two prediction tools and found to be involved in tumorigenesis, including in CRC [23,24]. To validate the specific binding between miR-381 and SPIN1, a mutated luciferase reporter vector was constructed as the mutant-type (MUT) SPIN1 3ʹ-UTR (Figure 3(a)). As clearly shown in Figures 3(b) and (c), miR-381 mimics or miR-381 inhibitor significantly affected the luciferase activity in CRC cells co-transfected with WT SPIN1 3ʹUTR, but not the MUT SPIN1 3ʹUTR. Moreover, qRT-PCR data showed that overexpression of miR-381 significantly inhibited the expression of SPIN1 mRNA in HCT116 and LOVO cells, while miR-381 inhibitors exerted the opposite effects (Figure 3(d)). Furthermore, the western blotting analysis revealed that miR-381 upregulation remarkably repressed SPIN1 protein expression, while miR-381 downregulation increased SPIN1 expression in CRC cells (Figure 3(e)). As well-documented, miRNAs often possess opposite expression patterns different from their target genes [25]. Therefore, we detected the expression level of miR-381 in 42 pairs of CRC tissues. As shown in Figure 3(f), miR-381 was frequently downregulated in CRC tissues (25/42, 59.5%). After statistically analyzing the pathological characteristics of these 42 patients (Table 3), we found that downregulation of miR-381 was significantly related to undesirable characteristics, such as tumor size (p = 0.046) and TNM stage (p = 0.037). Taken together, our findings strongly support that SPIN1 is a target of miR-381, and miR-381 is downregulated and negatively correlated with poorer clinical characteristics in CRC.

**Table 3.** The association between miR-381 expression and clinicopathological factors in patients with colorectal cancer

		miR-381 expression (%)		P value
Clinicopathological factors	N	low	high	
	42	25(59.5)	17(40.5)	
Age (years)				
≤55	17	10(58.8)	7(41.2)	0.939
>55	25	15(60.0)	10(40.0)	
Sex				
Female	15	8(53.3)	7(46.7)	0.542
Male	27	17(63.0)	10(37.0)	
Tumor Size (cm)				
≤5	17	7(41.2)	10(58.8)	0.046
>5	25	18(72.0)	7(28.0)	
Lymph node metastasis				
Negative	26	12(46.2)	14(53.8)	0.050
Positive	16	13(81.3)	3(18.7)	
TNM stage				
I–II	24	11(45.8)	13(54.2)	0.037
III–IV	18	14(77.7)	4(22.2)	
Differentiation				
Well-middle	26	14(53.8)	12(46.2)	0.339
Poorly	16	11(68.8)	5(31.2)	

All the data were analyzed by the chi-square test or Fisher exact test.

### MiR-381 overexpression impedes CRC cell proliferation and invasion

**Figure 4.**
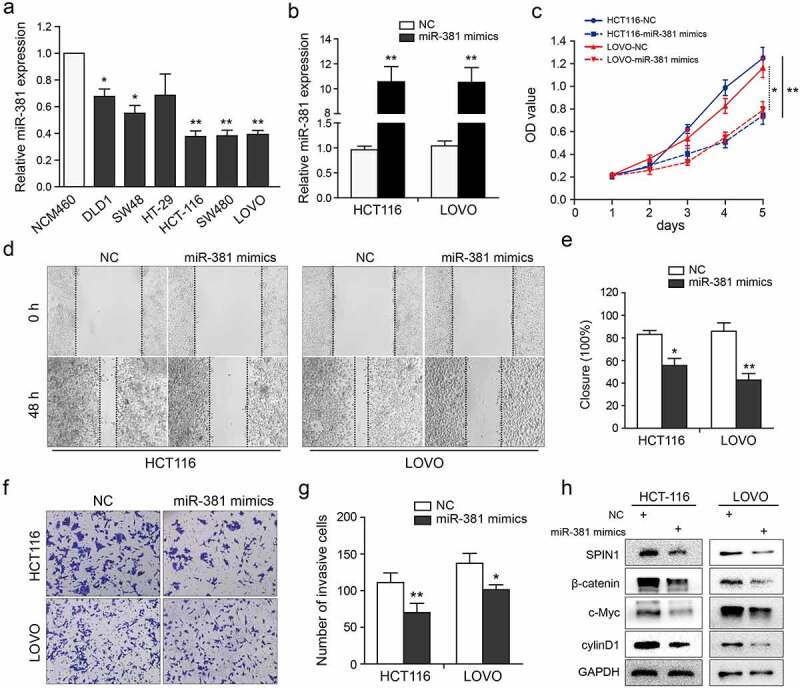
MiR-381 overexpression impedes CRC cell proliferation and invasion. (a) The miR-381 expression in CRC cell lines was evaluated using qRT-PCR assays. (b) Transfection deficiency was validated by qRT-PCR assays in HCT116 and LOVO cells. (c) The well growth abilities of HCT-116 and LOVO were assessed using CCK-8 assays after relative transfection. (d-g) The role of miR-381 on migration and invasion was detected by wound healing and transwell invasion assays. Representative figures (magnification, ×200) and quantification analysis were formed by the indicated groups of cells. (h) Representative western blotting images of SPIN1 and Wnt/β-catenin pathway components following indicated transfection. GAPDH was used as an internal control. Student’s t test, *p < 0.05, **p < 0.01.

We further investigated the biological role of miR-381 in CRC cells. As clearly shown in Figure 4(a), miR-381 expression in CRC cells was markedly decreased when compared to NCM460 cells. Then, the qRT-PCR assays were used to validate the transfection efficiency of miR-381 mimics or negative controls (Figure 4(b)). Clearly, miR-381 overexpression significantly suppressed cell growth, migration, and invasion abilities (Figure 4(c-g)), which were similar to the effects of SPIN1 depletion. In addition, miR-381 overexpression inhibited the activity of Wnt/β-catenin pathway (Figure 4(h)). Thus, these data indicate that miR-381 overexpression impedes cell proliferation and invasion of CRC cells.

### MiR-381 and SPIN1 are responsible for controlling the Wnt/β-catenin signaling pathway in CRC

**Figure 5.**
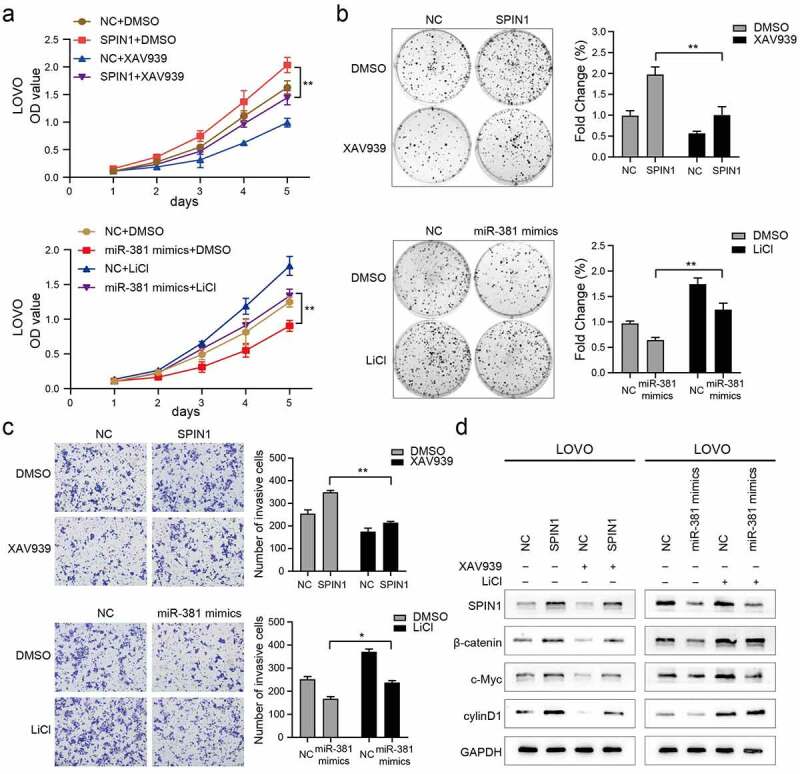
MiR-381 and SPIN1 are responsible for controlling the Wnt/β-catenin signaling pathway in CRC. (a-d) Rescue experiments of overexpressed SPIN1 and miR-381 mimics using with XAV939 or LiCl treatment for 24 h (XAV939,10 μmol/L; LiCl: 20 mmol/L). CCK-8 assays (a), Colony formation assays (b) and transwell invasion assay (c) were conducted to assess cell biological function upon indicated treatments. (d) The protein levels of SPIN1 and Wnt/β-catenin signaling pathway related targets (β-catenin, c-Myc and cyclin D1) were assessed by western blotting upon indicated treatments in LOVO cells. GAPDH was used as an internal control. Student’s t test, *p < 0.05, **p < 0.01.

To further confirm that miR-381 and SPIN1 are critical in regulating cell proliferation and invasion by orchestrating the Wnt/β-catenin signaling pathway, the Wnt/β-catenin pathway inhibitor or activator was added for reverse assays. As shown in Figure 5(a), XAV-939 markedly rescued the proliferation and viability of SPIN1-overexpression CRC cells. Similarly, LiCl significantly suppressed the proliferation of CRC cells transfected with miR-381 mimics. The same phenomenon was observed in the colony formation assays and transwell invasion assays (Figure 5(b,c)). More interestingly, we also observed that changes in Wnt/β-catenin pathway-related proteins (β-catenin, c-Myc, and cyclin D1) caused by SPIN1 overexpression plasmid or miR381 mimics can be recovered by XAV939 or LiCl, respectively (Figure 5(d)). Collectively, these results demonstrated that MiR-381 and SPIN1 are responsible for controlling the Wnt/β-catenin signaling pathway, thereby affecting cell proliferation and invasion in CRC cells.

### Ectopic expression of SPIN1 attenuates the miR-381-induced effects on CRC cells

**Figure 6.**
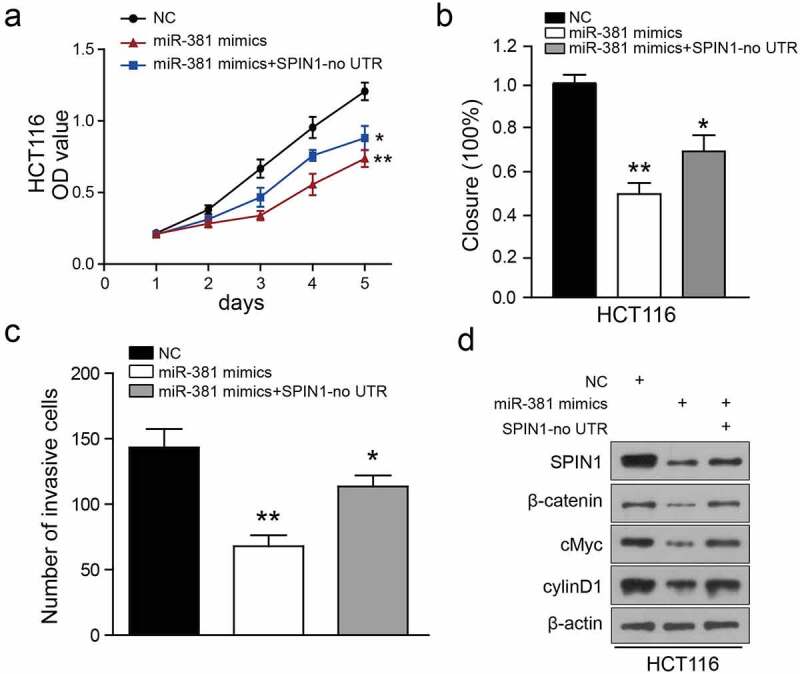
Ectopic expression of SPIN1 attenuates the miR-381-induced effects on colorectal cancer cells. (a) CCK-8 assays performed on miRNA-381-overexpressing HCT-116 cells with SPIN1 overexpression plasmid. (b) Wound healing assays performed on miR-381-overexpressing HCT-116 cells with SPIN1 upregulation. (c) Quantification of the transwell invasion assays under the indicated transfection condition. (d) Representative western blotting images of lysates prepared from miRNA-381-overexpressing HCT-116 cells with SPIN1 upregulation. One-way ANOVA test, *p < 0.05, **p < 0.01.

To investigate whether SPIN1 is a functional mediator of miR-381-induced effects, we synthesized a SPIN1 overexpression plasmid without 3ʹUTR and performed rescue assays. As illustrated in Figure 6(d), SPIN1 overexpression plasmid transfection restored SPIN1 expression, which was also partially suppressed by miR-381 overexpression in HCT116 cells. Similarly, functional experiments have demonstrated that overexpression of SPIN1 ameliorated the cancer-suppressive effects caused by miR-381 overexpression in CRC cells (Figure 6(a-c)). Surprisingly, overexpression of SPIN1 could not completely abolish the effects of miR-381 on the Wnt/β-catenin pathway in HCT116 cells (Figure 6(d)), suggesting that there may be other oncogenes or pathways involved in miR-381-mediated functions. In summary, these findings provide further evidence that SPIN1 functions as a main functional target of miR-381 in CRC.

## Discussion

CRC is the third leading cause of cancer-related mortality globally, with a heavy burden on society [26]. A detailed investigation of the underlying mechanism contributing to CRC development could provide new insights into the detection and treatment of CRC patients. Our previous data indicated that SPIN1 promotes tumorigenesis by negatively regulating the p53 pathway in multiple cancer cells [9]. In the present study, our data revealed that the miR-381/ SPIN1 axis is critical in CRC proliferation and invasion via controlling the Wnt/β-catenin pathway.

Mounting evidence has demonstrated that dysregulation of SPIN1 contributes greatly to cancer tumorigenesis and progression in a series of tumors [27–29]. Zhao et al. found that SPIN1 is highly expressed and triggers aberrant lipid metabolism in hepatocellular carcinoma patients [6]. Moreover, increased SPIN1 expression enhanced cell growth, invasion and cell cycle progression in gastric cancer cells [7]. Our study showed that SPIN1 was frequently upregulated in CRC tissues compared to adjacent noncancerous tissues. Additionally, SPIN1 upregulation was significantly associated with advanced clinical stage, depth of invasion, and metastasis, highlighting its critical role in the initiation and progression of CRC. Biological experiments have shown that SPIN1 depletion results in suppressed cell proliferation and invasion abilities in CRC cells, further implying that SPIN1 is a potential oncogene in CRC patients.

Numerous studies have documented that miRNAs are abnormally dysregulated in tumorigenesis, including CRC [30–34]. Recently, SPIN1 was found to be negatively regulated by miR-148/152, leading to adriamycin resistance in breast cancer [5]. Our luciferase reporter assays demonstrated that miR-381 could directly target the 3ʹ- UTR of SPIN1 mRNA. Several studies have suggested that miR-381 exerts its critical role in physical and pathological processes, including cell growth, apoptosis and tumorigenesis [35–39]. For example, dendrobine suppressed the endoplasmic reticulum stress-induced apoptosis by increasing miR-381-3p expression in endothelial cells [38]. Previously, miR-381 was reported to suppress cell proliferation and metastasis by targeting the BM1 and Rho/ROCK axis in bladder cancer [40]. Additionally, miR-381 facilitates autophagy and apoptosis by suppressing the RELN-mediated PI3K/Akt pathway in prostate cancer cells [41]. However, miR-381 may also exert oncogenic functions in some other cancers, including epithelioid sarcoma and glioma [42,43]. In the present study, we further strengthened the tumor suppressive role of miR-381 in CRC cells. Our data showed that miR-381 was frequently decreased in CRC clinical samples and cell lines. Moreover, functional assays revealed that overexpression of miR-381 repressed cancer cell proliferation and invasion, which recapitulated the impact of SPIN1 depletion. These data indicate that miR-381 exerts its tumor-suppressive roles by inhibiting SPIN1 expression, and this newly identified miR-381/SPIN1 regulatory axis greatly contributes to CRC tumorigenesis and progression.

It is well-established that the excessive activation of the Wnt/β-catenin pathway is involved in the development in CRC cells [44]. Previous studies have shown that SPIN1 serves as a positive modulator of Wnt/β-catenin pathway [4,45]. In line with this, our study found that Wnt target genes, c-Myc and cyclinD1, were regulated by SPIN1. Moreover, β-catenin is also regulated by the level of SPIN1. We suspect SPIN1 overexpression might attract β-catenin translocates from the cytoplasm to the nucleus, thereby avoiding β-catenin degradation in the cytoplasm. In addition, our previous data showed that miR-381 delayed cancer progression in gastric cancer by suppressing the Wnt/β-catenin pathway [35]. Consistent with it, we also found that miR-381 upregulation impeded the malignant behaviors of CRC cells and reintroduction of SPIN1 rescued the inhibitory effect of miR-381 on the Wnt/β-catenin pathway. More interestingly, the Wnt/β-catenin signaling-specific inhibitor XAV-939 and activator LiCl partially eliminated the effects of SPIN1 overexpression and miR-381 mimics, which further supports the notion that the miR-381/SPIN1 axis is critical for regulating the Wnt/β-catenin pathway. Besides, it is worth considering that SPIN1 overexpression could not completely abolish miR-381-induced effects, indicating that other mechanisms may also participate in miR-381 functions. In summary, our data indicate that miR-381 restrains the activation of the Wnt/β-catenin pathway by targeting SPIN1.

## Conclusions

Collectively, our results showed that the dysregulated miR-381/SPIN1 axis greatly contributes to the initiation and progression of CRC cells by orchestrating the Wnt/β-catenin pathway, and therefore represents an actionable therapeutic target for CRC patients.

## Data Availability

The datasets used or analyzed during the present study are available from the corresponding authors Ziling Fang and Xiaojun Xiang upon reasonable request.

## Highlights

- SPIN1 is frequently upregulated in CRC, and SPIN1 overexpression facilitates the cell proliferation and invasion of CRC cells.
- SPIN1 is a target of miR-381.
- MiR-381 impedes colorectal cancer cell proliferation and invasion through inactivation of the Wnt/β-catenin pathway via targeting SPIN1.
